# The complete chloroplast genome of *Actinidia rubus* (Actinidiaceae)

**DOI:** 10.1080/23802359.2019.1703571

**Published:** 2020-01-07

**Authors:** Yu-Sheng Xu, Cheng-Gui Zhang, Jun-Tong Chen, Li-Juan Li, Chuan-Jiang Liao, Zhen-Yu Lv, Zhi-Lin Jiang

**Affiliations:** a Institute of Entomoceutics Research, National-Local Joint Engineering Research Center of Entomoceutics, Dali University, Dali, China;; b Key Laboratory for Plant Diversity and Biogeography of East Asia, Kunming Institute of Botany, Chinese Academy of Sciences, Kunming, China;; c Key Laboratory of Plant Germplasm Enhancement and Specialty Agriculture, Wuhan Botanical Garden, Chinese Academy of Sciences, Wuhan, China;; d School of Life Sciences, Yunnan Normal University, Kunming, China;; e Institute of Agricultural and Garden Technology, Puer University, Puer, China;; f Institute of Comparative Study of Traditional Materia Medica, Institutes of Integrative Medicine, Fudan University, Shanghai, China

**Keywords:** *Actinidia rubus*, chloroplast genome, phylogenetic analysis

## Abstract

A complete chloroplast genome of *Actinidia rubus*, an endemic shrub in China, was sequenced and identified. The length of genome is 156,573 bp, and the GC content is 37.3%. This genome contains a large single copy (LSC; 88,473 bp) region, a small single copy (SSC; 20,492) region, a pair of inverted repeat (IR; 23,804) regions. A total of 113 unique genes were identified, including 78 protein-coding genes, 31 tRNA genes and 4 rRNA genes. The phylogenetic analysis based on complete chloroplast genome of 10 species showed that *Actintdia eriantha* was sister to *A. rubus*.

*Actinidia rubus*, an endemic species of *Actinidia* in China, was found only in Yunnan and Sichuan provinces. It is a deciduous climbing shrub with densely reddish brown strigose petiole and irregularly setose-serrulate leaf margin (Li et al. 2003). Until now, the complete chloroplast genome of *A. rubus* has not been reported, even few records of this species have been discovered. The complete chloroplast genome of *A. rubus* will benefit the study of *Actinidia*. Therefore, we sequenced and assembled the complete chloroplast genome of *A. rubus* based on high-throughput sequencing and reconstructed the phylogenetic tree to determine its phylogenetic position in this study.

The voucher specimens were stored in KUN (i.e. Herbarium, Kunming Institute of Botany, CAS; KUN1347950). Fresh leaves of *A. rubus* were collected from Wumeng Mountian, Yongshan County, Yunnan, China (28.25165555555555°N, 103.9791305555556°E). The genome of *A. rubus* was isolated and sequenced using Illumina sequencing methods at the Beijing Novogene Bioinformatics Technology Co., Ltd. NOVOPlasty v.3.3 (Dierckxsens et al. 2017) was used to assemble the filtered reads with the referenced complete chloroplast genome of *Actinidia chinensis* (GenBank accession no. KP297243.1). The assembled plastome was annotated using Geseq at Chlorobox web service (Tillich et al. 2017), and the result of annotation was manually corrected using Geneious v.9.0.2 (Kearse et al. 2012). At last, the complete chloroplast genome of *A. rubus* (MN652056) with annotations has been submitted to Genbank.

The complete chloroplast genome of *A. rubus* is 156,573 bp in size, which has a typical quadripartite structure, including a LSC region of length 88,473 bp, a SSC region of length 20,492 bp, two IR regions of 23,804 bp in length. The overall GC contact is 37.3%. In total, 131 genes were annotated, and 113 unique genes were identified from among them, including 78 protein-coding genes, 31 tRNA genes and 4 rRNA genes. Most of unique genes are single copy, but there are 4 rRNA genes (*rrn4.5*, *rrn5*, *rrn16*, *rrn23*), 10 tRNA genes (*trnH-GUG*, *trnI-CAU*, *trnL-CAA*, *trnV-GAC*, *trnI-GAU*, *trnA-UGC*, *trnR-ACG*, *trnN-GUU*, *trnT-GGU*, *trnM-CAU)*, 4 protein-coding genes (*ycf2*, *ycf15*, *ndhB*, *rps7*) with double copies. In addition, 15 genes contain intron, one of which has two introns (*ycf3*).

The phylogenetic analysis of *A. rubus* was based on its whole chloroplast genome sequence and that of other nine species of Actinidiaceae. All genome sequences were multiple aligned using MAFFT v.7.308 (Katoh et al. 2002). The maximum-likelihood (ML) phylogenetic tree was constructed using IQtree v.1.6.12 with 1000 bootstrap replicates (Trifinopoulos et al. 2016). The results were consistent with previous study (Zhang and Liu 2019), which showed *Actintdia eriantha* was sister to *A. rubus* with 98% bootstrap values (Figure 1).

**Figure 1.**
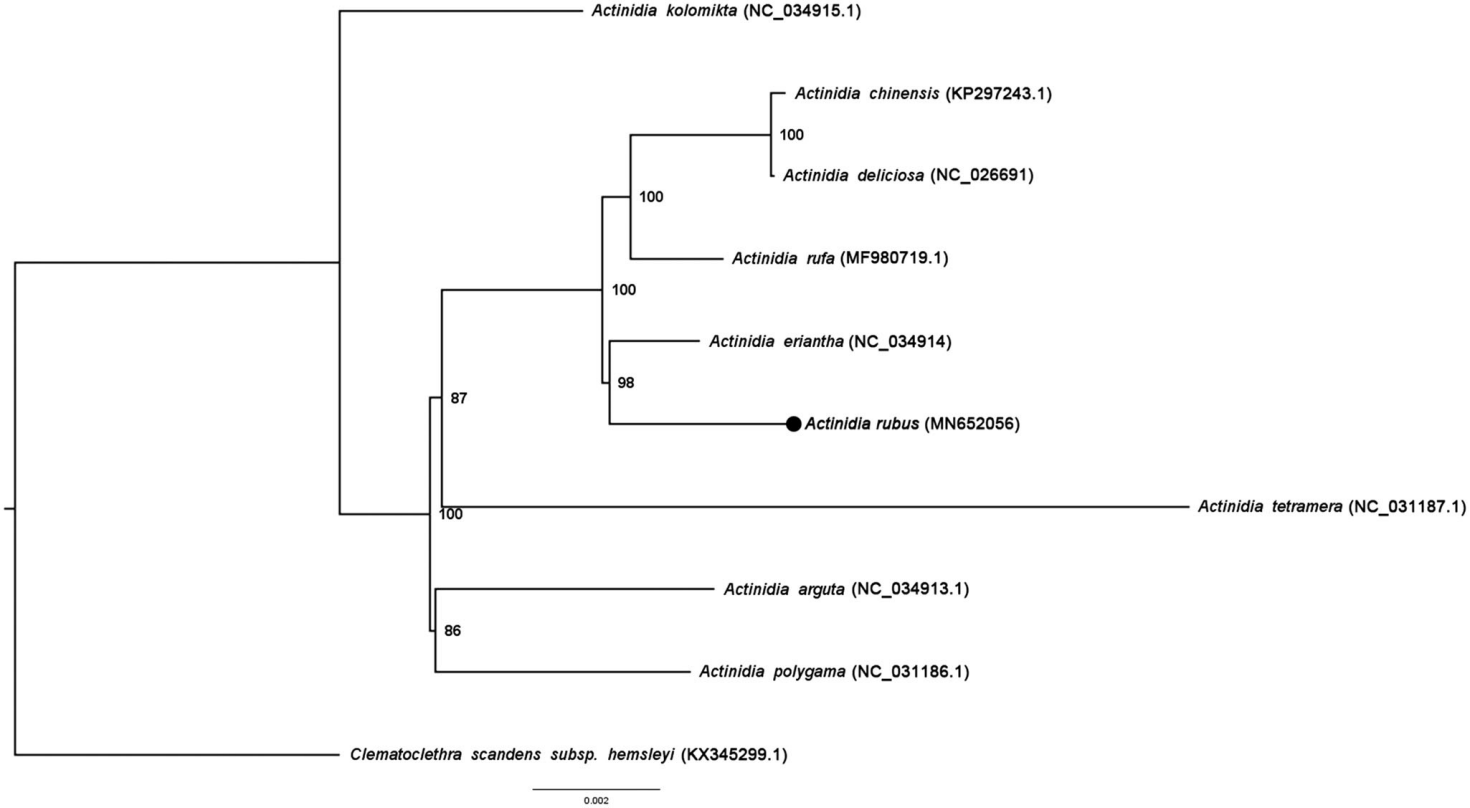
Phylogeny of 10 species based on complete chloroplast genome using maximum-likelihood Estimate. The numbers at the nodes are bootstrap values. The black dot indicates *Actinidia rubus*.
